# Using Wearable Device and Machine Learning to Predict Mood Symptoms in Bipolar Disorder: Development and Usability Study

**DOI:** 10.2196/66277

**Published:** 2025-09-16

**Authors:** Chia-Tung Wu, Ming H Hsieh, I-Ming Chen, Lian-Yin Jhao, Ding-Shan Liu, Ssu-Ming Wang, Chia-Ting Wu, Yi-Ling Chien

**Affiliations:** 1 Master Program in Transdisciplinary Long-term Care and Management, National Yang Ming Chiao Tung University, Taipei, Taiwan; 2 Department of Psychiatry, National Taiwan University Hospital, No.7, Chung-Shan South Road, Taipei, 10002, Taiwan, 886 2-23123456 ext 266013; 3 Always Support Technology Co, Ltd, New Taipei, Taiwan; 4 Department of Computer Science and Information Engineering, National Taiwan University, Taipei, Taiwan; 5 Graduate Institute of Biomedical Electronics and Bioinformatics, National Taiwan University, Taipei, Taiwan

**Keywords:** bipolar disorder, wearable device, machine learning, mood symptoms, relapse prediction

## Abstract

**Background:**

Bipolar disorder (BD) is a highly recurrent disorder. Early detection, early intervention, and prevention of recurrent bipolar mood symptoms are key to a better prognosis.

**Objective:**

This study aims to build prediction models for BD with machine learning algorithms.

**Methods:**

This study recruited 24 participants with BD. The Beck Depression Inventory and Young Mania Rating Scale were used to evaluate depressive and manic episodes, respectively. Using digital biomarkers collected from wearable devices as input, 6 machine learning algorithms (logistic regression, decision tree, k-nearest neighbors, random forest, adaptive boosting, and Extreme Gradient Boosting) were used to build predictive models.

**Results:**

The prediction model for depressive symptoms achieved 83% accuracy, an area under the receiver operating characteristic curve (AUROC) of 0.89, and an *F*_1_-score of 0.65 on testing data. The prediction model for manic symptoms achieved 91% accuracy, an AUROC of 0.88, and an *F*_1_-score of 0.25 on testing data. With the interpretable model Shapley Additive Explanations, we found that relatively high resting heart rate, low activity, and lack of sleep may predict depressive symptoms.

**Conclusions:**

This study demonstrated that digital biomarkers could be used to predict depressive and manic symptoms. This prediction model may be beneficial for the early detection of mood symptoms, facilitating timely treatment and helping to prevent BD recurrence.

## Introduction

Bipolar disorder (BD) is a recurrent disorder characterized by fluctuations in mood and energy from depression to mania that often results in enormous functional impairment and high disease burden [1]. The 5-year recurrence rate of BD was as high as 73% [2]. A meta-analysis estimated the recurrence rates under treatment were 55.2% (naturalistic studies) and 39.3% (randomized controlled trials) versus 60.6% under placebo [3]. Although the recurrence rate can be reduced under appropriate treatment, irregular compliance usually precipitates the recurrence of mood episodes and compromises the outcome. Despite high recurrence and low compliance, the frequency of routine follow-up in current practice is usually insufficient to detect early signs of relapse, causing delayed treatment until an acute episode has fully developed [4]. It is important to detect early signs of relapse so that an upcoming mood episode can be aborted by appropriate intervention. Furthermore, clinical practice mostly relies on patients’ self-reports of symptom changes, which depend on illness insight [5] and are subject to recall bias [6].

Recent studies have developed machine learning algorithms to predict depression [7] based on sociodemographic features, personal or family health history, and symptoms coded by psychiatrists [8], achieving an accuracy of 0.721 using the k-nearest neighbor classifier. In BD, a recent review concluded that activity level may serve as a promising digital phenotype for mood episodes, with decreased activity indicating depressive episodes and increased activity indicating manic episodes [9]. For example, Jakobsen et al [10] collected activity data through actigraphy to differentiate between depressed patients and controls, achieving an accuracy of 0.84 using the deep neural network combined with the Synthetic Minority Over-Sampling Technique (SMOTE) class balancing technique [10]. Besides, sleep problems may indicate relapse in BD [11]. A recent study that used only sleep-wake data gathered through smartphones and wearables showed accurate next-day predictions of depressive, manic, and hypomanic episodes (area under the receiver operating characteristic curve [AUROC] of 0.80, 0.98, 0.95, respectively) [12], suggesting sleep as a promising biomarker for detecting mood episodes. However, most current studies targeted full-blown mood episodes and followed patients for shorter time periods, and information about feature importance was often lacking.

Apart from activity level and sleep, heart rate (HR) and HR variability could also be important biomarkers for mood episodes. HR variability, defined as temporal variability in beat-to-beat intervals of HR, is supposed to reflect aspects of parasympathetic control over the cardiac system [13]; while HR was an index of sympathetic and parasympathetic influences of the autonomic nervous system during stressful as well as resting and recovery states [14]. During rest, HR is under central inhibitory control by the vagus nerve and thus decreased [15,16]. Increased HR and decreased HR variability were observed in mania relative to euthymia [17]. Likewise, patients with depression not only showed lower HR variability but also had a higher resting HR [18-20]. A study gathered biofeedback data on HR variability features and used support vector machine algorithms to predict mood state, reporting an accuracy of 0.69 [21]. Another machine learning study with HR variability features achieved an accuracy of 0.74 [22]. Considering future applications, we adopted HR features instead of HR variability in this study, since HR is much more commonly recorded in commercial smartwatches than HR variability.

Combining multimodal data sources of physical activity, sleep patterns, and circadian rhythms for a machine learning algorithm, a recent study reported an accuracy of 0.80 in detecting participants at high risk of depression [23]. Another study performed a prospective observational cohort study with patients with mood disorders, collecting activity, sleep, light exposure, and HR to predict mood state, and found the accuracy of 0.87, 0.94, and 0.91, respectively, for depressive episode, manic episode, and hypomanic episode [24]. Although both studies used multiple sources of physiological data, the feature importance and the direction of effect of the features were not clear. Besides, individual differences were not considered among the features.

Based on prior research, this study aimed to establish machine learning algorithms to predict early signs of upcoming depressive or manic symptoms. Digital biomarkers collected by actigraphy and mobile devices, including activity, sleep hours, and HR, were mapped with clinical measures for training and evaluating the machine learning models of artificial intelligence algorithms. We hypothesized that greater activity level and shorter sleep hours may predict manic symptoms. We also hypothesized that higher resting HR and lower activity level and sleep hour changes may predict depressive symptoms based on previous reports [18-20]. Moreover, considering that the change of a feature (eg, sleep or activity) from the individual’s baseline may be important when predicting mood symptoms, we also integrated individualized parameters for model prediction.

## Methods

### Participants

The inclusion criteria were participants with a diagnosis of BD, aged 20‐65 years, and willing to wear the smartwatch and complete the clinical measures as frequently as possible (at least weekly). The exclusion criteria were concurrent substance use disorder and inability to cooperate with the data collection from the smartphone and smartwatch.

This study recruited 24 patients with BD (aged 20‐65 years; mean 38, SD 9 years; male n=9) from the Psychiatric Department of National Taiwan University (NTU) Hospital from October 2020 to July 2022 and prospectively followed for an average of 6.39 (SD 4.85) months. The diagnosis was made by board-certified senior psychiatrists based on the *Diagnostic and Statistical Manual of Mental Disorders, Fifth Edition* [25]. The mean age of first onset of mood episodes was 23.1 (SD 10) (range 12‐44), and the illness duration was 14.1 (SD 8.7) years. During the follow-up, there were a total of 4 psychiatric admissions. All participants received medication treatment, primarily mood stabilizers and antipsychotics (Table S1 in Multimedia Appendix 1). Participants’ educational levels ranged from middle school to graduate school (7 graduate school degrees, 13 college, 3 senior high school, and 1 junior high school). As for medical history, all participants had no active medical disease, but with a history of Sicca syndrome (n=1), cavernous sinus cyst (n=1), atrial septal defect (n=1), and one peptic ulcer (n=1).

### Clinical Measures

The Beck Depression Inventory (BDI) and Young Mania Rating Scale (YMRS) were self-rated weekly on a mobile app (named MEDGOD) (National Taiwan University) on the personal smartphone (either iOS or Android system) to evaluate depressive and manic symptoms, respectively. All participants received a reminder notification on Sunday night at 9 PM to fill both measures on their mobile app. The research assistants would send a text message, email, or phone call to contact the participants and help solve technical problems.

The BDI [26] is a 21-item self-reported inventory measuring the severity of depression in adolescents and adults. The Beck Depression Inventory-II (BDI-II) [27] was revised to be more consistent with the *DSM-IV* (*Diagnostic and Statistical Manual of Mental Disorders, Fourth Edition*) criteria of depression. The inventory consists of 21 items, in which 4 response options are presented on a Likert scale from 0 to 3. The BDI was translated into a Chinese version with good internal consistency (Cronbach α=0.85) and concurrent validity [28]. Higher total scores indicate more severe depressive symptoms. As for the standard cutoff scores, 30‐63 may indicate severe depression, 19‐29 indicates moderate depression, 10‐18 indicates mild depression, and 0‐9 indicates minimal depression [28]. The distribution of BDI scores in our sample was summarized in Multimedia Appendix 2.

The YMRS, an 11-item interviewer-rated scale, is designed for assessing the severity of manic symptoms [29]. The items are rated on 5 grades of severity. Four items among them are double weighted, including irritability, speech, thought content, and disruptive or aggressive behavior. The YMRS is by far the most commonly used standardized measure of bipolar manic symptoms for clinical trials in acute mania. As for psychometric properties, the interrater reliability was adequate for the total score (0.93) and for individual items ranged from 0.67 to 0.95. There are no firmly established scoring criteria that relate to diagnostic classification [29]. The distribution of YMRS scores in our sample was summarized in Multimedia Appendix 3.

To detect early signs of relapse, a state of mild depression (BDI >13) or mania (YMRS >13) was labeled true.

### Digital Biomarkers

The wristwatch-like actimetry sensor Garmin Vivosmart 4 is a reliable tool [30,31] that has been applied in several clinical studies [32-34]. Each participant wore the smartwatch that continuously measured as well as recorded the motor activity, sleep length, and HR 24 hours a day except when electric recharging.

The motor activities that were monitored included steps, distance traveled, and floors climbed. The sleep features included total sleep hours and stages of sleep, that is, deep sleep, light sleep, rapid eye movement sleep, and awake stages. The HR features included the minimum HR of the day, the maximum HR of the day, the average HR of the day, and the average HR at rest.

As for individualized features, we transformed the raw feature into “the deviation from the personal mean” by subtracting the raw value by the mean of that feature of the person during the entire study period, including differences in minimum HR, maximum HR, average HR, resting HR, steps, distance, and the total sleep (as listed in Tables S2 and S3 in Multimedia Appendix 1).

### Data Pretreatment

Data were initially uploaded to the server of NTU Medical Genie Precision Health Service via the NTU MEDGOD app automatically. Subsequently, the information was downloaded and stored at the National Taiwan University Computer and Information Networking Center, which provides optimized information security protection [35]. The digital data were deidentified, managed, and maintained by the Medical Information Laboratory of the Department of Information Engineering, National Taiwan University, as described in our previous studies [33,36,37].

**Figure 1.**
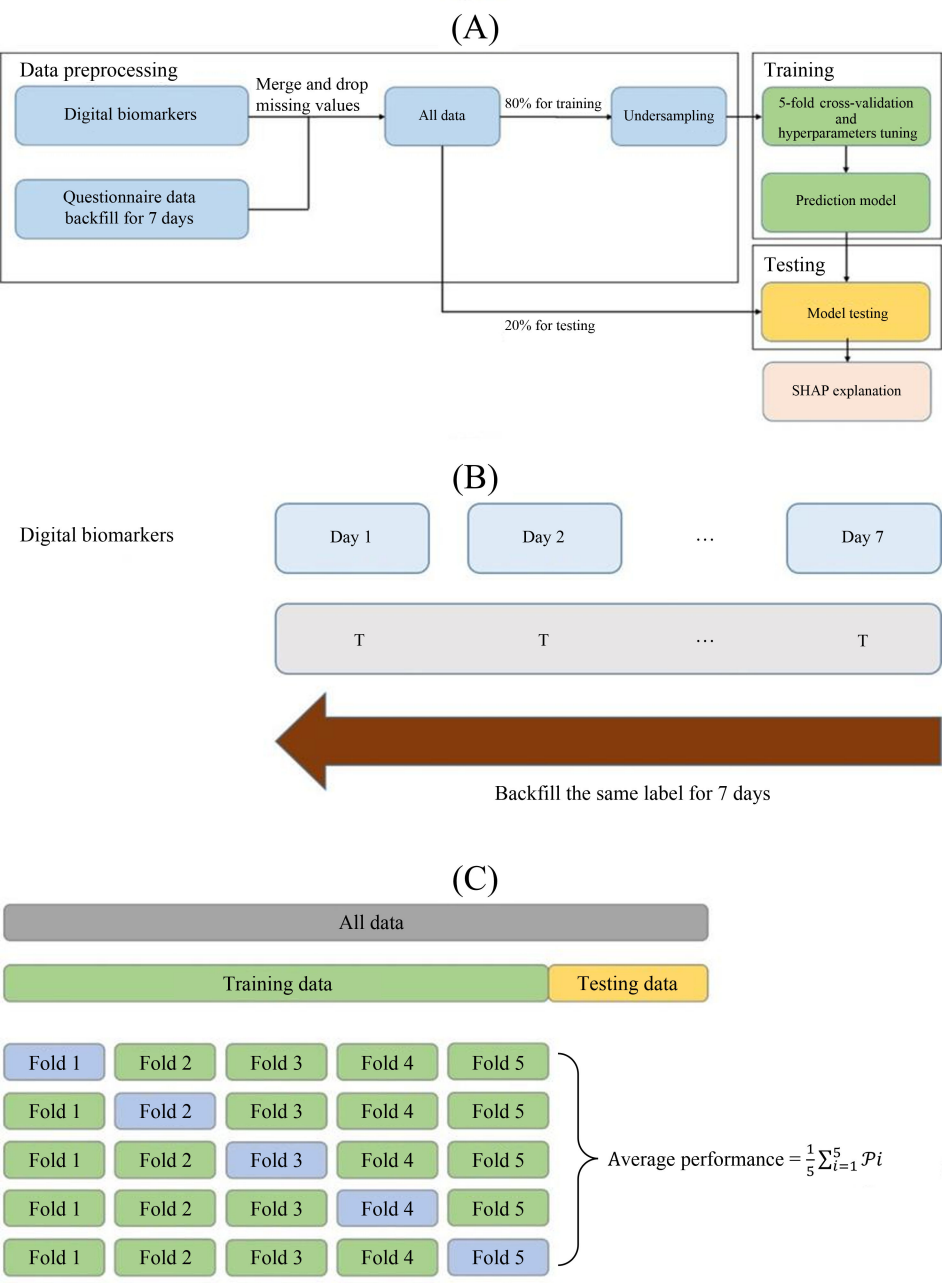
Workflow architecture (A), backfill structure (B), and *5*-fold cross-validation (C). SHAP: Shapley Additive Explanations.

The YMRS and BDI-II were administered weekly for the mood-related symptoms of the previous week; thus, the physiological data were backfilled for a 7-day period (Figure 1B). To mitigate the risk of overfitting, all missing values were excluded from the dataset. Extreme datapoints, such as HR>150 or <40, total sleep hours >15, were excluded from analysis, too. Subsequently, the dataset was partitioned into training and testing sets using 5-fold cross-validation.

Figure 1A shows the workflow architecture including interpretable model Shapley Additive Explanations (SHAP). To achieve more balanced data, the under-sampling technique [38] was applied for the training set.

### Machine Learning Algorithms

Depressive and manic models were trained separately using the first half of the follow-up data. Python, scikit-learn, and SHAP packages were used for programming, model training, and model explanation, respectively. We adopted logistic regression, decision tree, k-nearest neighbors, random forest, adaptive boosting, as well as Extreme Gradient Boosting (XGBoost) to predict mood symptoms. Five-fold cross-validation was used to evaluate model performance and hyperparameter selection before making predictions on testing data [39] (Figure 1C). During the training process, the grid search strategy for hyperparameter selection was used to ensure model stability, which loops through all candidate parameters, leaving only the final best-performing set of parameters.

### Model Assessment

Model performance was assessed by accuracy, sensitivity, specificity, precision, *F*_1_-score, and AUROC. To explain the output of machine learning models [40], the SHAP method analyzed the predictions from machine learning models and interpreted the contributions of each feature by a Shapley value, which measures the influence of a feature on the prediction.

### Ethical Considerations

This study was approved by the Research Ethics Committee at National Taiwan University Hospital (202002006RINA) before its implementation. The investigation was carried out in accordance with the latest version of the Declaration of Helsinki. Informed consent of the participants was obtained after the nature of the procedures had been fully explained. Data were anonymized. Participants who completed the follow-up received a small gift (ie, essential oil soap) valued at less than US $6.00.

## Results

### Overview

The features were compared between the depressive and nondepressive labels, and between the manic and nonmanic labels by Student *t* test (Tables S2 and S3 in Multimedia Appendix 1). We found that the depressive label had a significantly higher minimal or resting HR and a lower activity level than the nondepressive label. Besides, the manic label had a significantly lower sleep duration than the nonmanic label. Similarly, for individualized data, we found that the manic label had a significantly higher HR and activity level and lower sleep duration than the nonmanic label.

### Prediction Model

First, we used 12 features (without individualized data) for model training. Table 1 shows model performance on the testing set. Compared to other algorithms, the XGBoost performed with the highest accuracy (0.79), AUROC (0.85), and *F*_1_-score (0.57) in the depressive model. Similarly, in the manic model, the XGBoost performed superior to others, with the highest accuracy (0.83), AUROC (0.84), and *F*_1_-score (0.19).

**Table 1.** The performance of the models for depressive or manic symptoms without individualized features in the models.

	Accuracy	AUROC^a^	Sensitivity	Specificity	Precision	*F*_1_-score
Models for a depressive episode						
Logistic regression	0.63	0.77	0.88	0.57	0.34	0.49
Decision tree	0.74	0.65	0.34	0.84	0.35	0.35
KNN^b^	0.69	0.58	0.38	0.77	0.30	0.34
Random forest	0.70	0.83	0.87	0.66	0.39	0.54
AdaBoost^c^	0.78	0.79	0.40	0.87	0.44	0.42
XGBoost^d^	0.79	0.85	0.71	0.81	0.48	0.57
Models for a manic episode						
Logistic regression	0.65	0.63	0.58	0.65	0.05	0.09
Decision tree	0.87	0.64	0.25	0.89	0.07	0.10
KNN	0.90	0.51	0.08	0.93	0.03	0.05
Random forest	0.75	0.78	0.58	0.75	0.07	0.12
AdaBoost	0.94	0.74	0.08	0.97	0.08	0.08
XGBoost	0.83	0.84	0.67	0.84	0.11	0.19

a AUROC: area under the receiver operating characteristic curve.

b KNN: k-nearest neighbors.

c AdaBoost: Adaptive Boosting.

d XGBoost: Extreme Gradient Boosting.

Next, we examined whether adding individualized features to the models improved prediction. The prediction performance of the 19-feature model with individualized features was successfully improved in both depressive and manic models (Table 2). The accuracy, AUROC, and *F*_1_-score in the depressive model by the XGBoost increased from 0.79, 0.85, and 0.57 to 0.83, 0.89, and 0.65, while those in the manic model increased from 0.83, 0.84, and 0.19 to 0.91, 0.88, and 0.25.

**Table 2.** The performance of the models for depressive or manic symptoms with the individualized features included in the models.

	Accuracy	AUROC^a^	Sensitivity	Specificity	Precision	*F*_1_-score
Models for a depressive episode						
Logistic regression	0.86	0.87	0.62	0.92	0.68	0.65
Decision tree	0.79	0.75	0.50	0.87	0.49	0.50
KNN^b^	0.79	0.70	0.54	0.85	0.48	0.51
Random forest	0.82	0.86	0.79	0.83	0.55	0.65
AdaBoost^c^	0.82	0.83	0.54	0.89	0.56	0.55
XGBoost^d^	0.83	0.89	0.78	0.85	0.56	0.65
Models for a manic episode						
Logistic regression	0.71	0.84	0.92	0.71	0.09	0.16
Decision tree	0.90	0.69	0.42	0.92	0.14	0.20
KNN	0.89	0.62	0.33	0.91	0.10	0.15
Random forest	0.89	0.81	0.25	0.91	0.08	0.12
AdaBoost	0.93	0.78	0.33	0.95	0.16	0.22
XGBoost	0.91	0.88	0.50	0.92	0.17	0.25

a AUROC: area under the receiver operating characteristic curve.

b KNN: k-nearest neighbors.

c AdaBoost: Adaptive Boosting.

d XGBoost: Extreme Gradient Boosting.

### Explanation of Prediction Model

**Figure 2.**
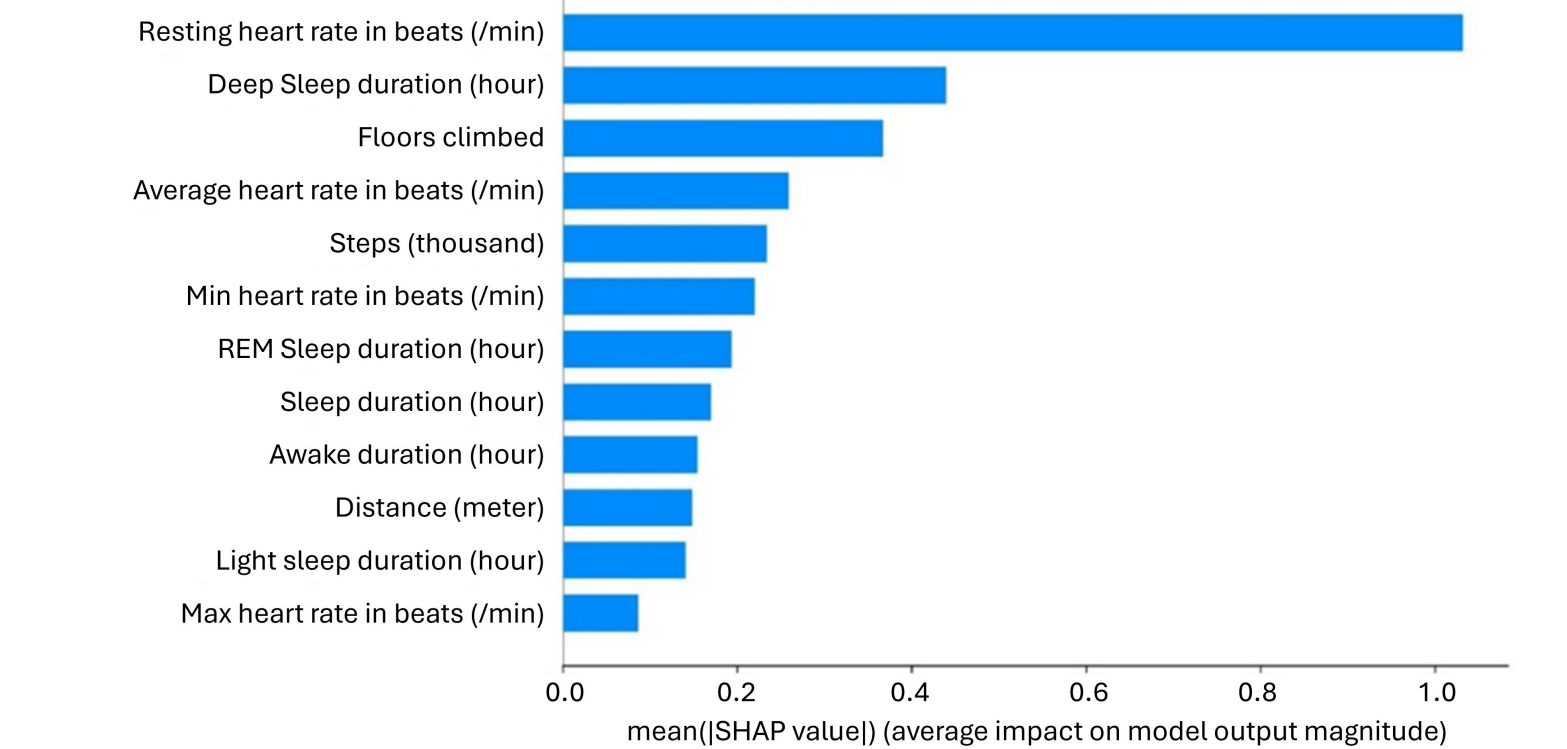
Feature importance of Extreme Gradient Boosting (XGBoost) with the summary plot. REM: rapid eye movement; SHAP: Shapley Additive Explanations.

**Figure 3.**
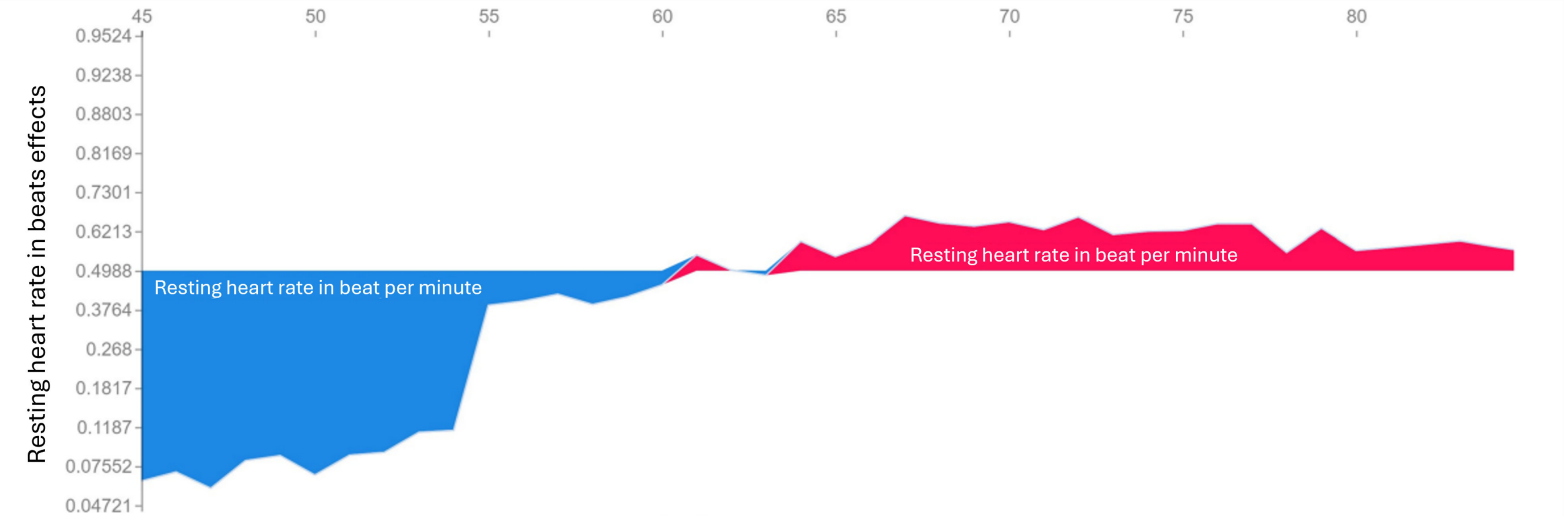
Impact of resting heart rate on the model with the force plot. The x-axis of the figure is the number of resting heartbeat per minute, and the y-axis represents the magnitude of the impact on the model.

**Figure 4.**
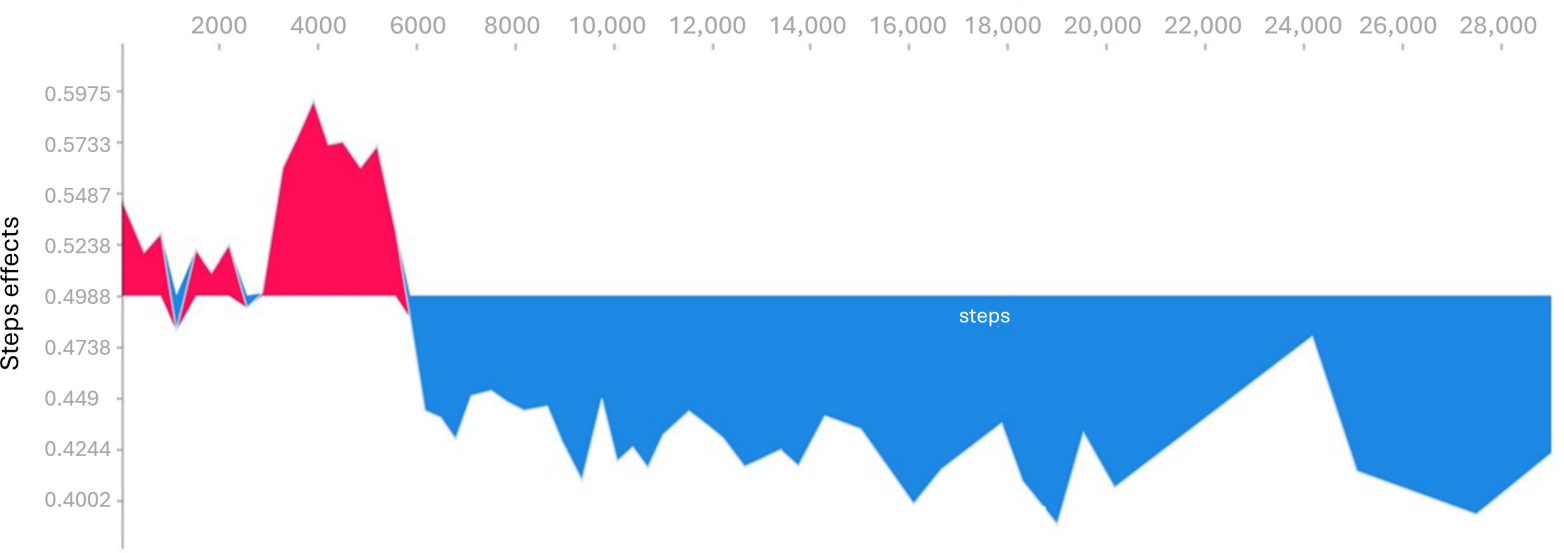
Impact of steps on the model with the force plot. The x-axis of the figure is the number of steps per day, and the y-axis represents the magnitude of the impact on the model.

**Figure 5.**
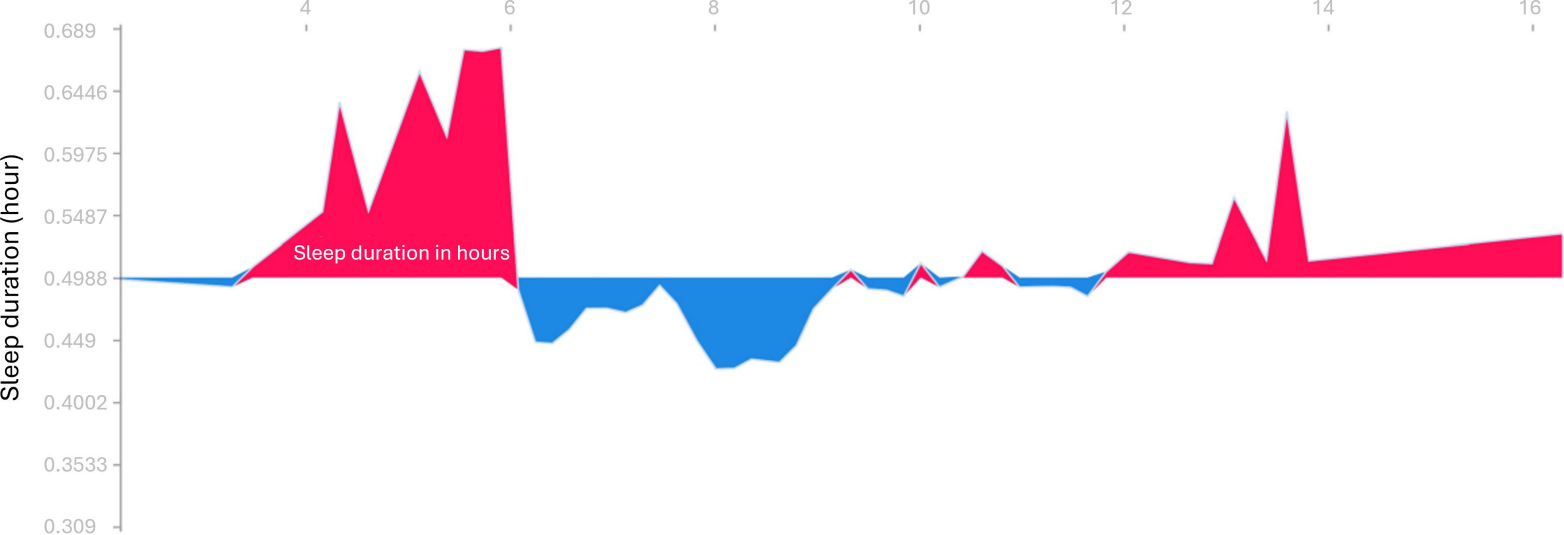
Impact of total sleep duration in hours on the model with the force plot. The x-axis of the figure is the number of total sleep duration per day, and the y-axis represents the magnitude of the impact on the model.

**Figure 6.**
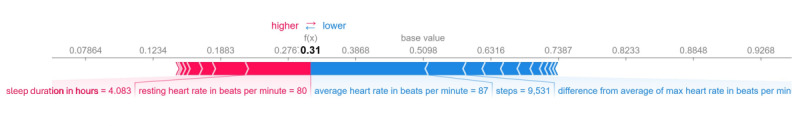
Impact of different features on a single data point with the force plot.

In the depressive model, resting HR revealed the highest feature importance, followed by deep sleep duration, floors climbed, average HR, and steps (Figure 2). The force plots explained how resting HR (Figure 3), number of steps (Figure 4), and total sleep duration (Figure 5) affect the model. In each force plot, the y-axis represents the magnitude of the impact on the model, where a positive value (red region) indicates a tendency to predict that there are mood symptoms of depression, and a negative value (blue region) suggests the opposite. We could see that there is an obvious turning point at 60 in Figure 3. Specifically, steps less than 6000 steps/day and sleep less than 6 h may predict depressive symptoms. Together, a high resting HR produces a push to the right, while sufficient activity and sleep produce a push to the left (Figure 6); all forces result in a 0.23 predicted probability of a depressive episode.

## Discussion

### Principal Findings

This study constructed prediction models for mild depressive and manic symptoms using digital biomarkers, including activity, sleep state, HR, and the individualized parameters of these features. Major findings included, first, the accuracy of the prediction achieved 79% (AUROC 0.85) in the depressive model and 83% (AUROC 0.84) in the manic model. Second, adding individualized features improved accuracy to 83% (AUROC 0.89) in the depressive model and 91% (AUROC 0.88) in the manic model. Third, among the features, higher resting HR, lower activity, and shorter sleep were important in predicting depressive symptoms. These findings supported the utility of digital biomarkers collected from wearable devices in predicting depressive and manic symptoms in the early phase.

The accuracy and AUROC in our depressive model (0.84) were similar to an earlier study in mood disorder (0.87) [24], with a higher sensitivity in ours (0.78 vs 0.48) yet higher specificity in theirs (0.96) than ours (0.85). Also, the accuracy (ours 0.84 vs 0.80), sensitivity (ours 0.78 vs 0.82), and specificity (ours 0.85 vs 0.78) were close to a previous multimodal source data algorithm for depression [23]. In the manic model, their accuracy (0.91) and AUROC (0.91) were close to ours, but their sensitivity (0.31) was lower than ours (~0.92) and their specificity (0.996) was higher than ours (0.71). Such discrepancy may be related to the machine learning algorithm (random forest in theirs vs XGBoost in ours) and individualized features included in our model. Higher sensitivity in our model may indicate a potential application in detecting symptoms in the early stage. Whereas a relatively lower specificity may necessitate a thorough clinical assessment after a case has been identified. In addition, they provided model performance and feature importance, but we further explained the model by using insights from SHAP to explain the clinical implications of the features. Of note, the mean ages and first onset of mood disorder were 25.9 (SD 4.8) and 17.9 (SD 4.8) in their study and 38 (SD 9) and 23.1 (SD 10) in our study. Given that the HR, sleep, and activity levels may depend on age, the older sample in ours may also contribute to the discrepancies of findings across the studies. The age effect on the predictive model is worth further exploration.

Our findings supported the prediction of depressive symptoms by digital features including activity level, sleep parameters, and HRs during the 7 days before depressive symptoms were reported. Among the features, we found that a higher resting HR may contribute to the prediction of depression. Previous studies also showed that the resting HR was higher in depressive patients [14-16]. Severe depression is often accompanied by increased HR [41] and reduced HR variability [41,42]. Our data suggested a turning point of resting HR at 60 (Figure 6), showing that resting HR higher than 60 was linked to a higher chance of predicting depressive symptoms. Depressive disorders have been shown to be associated with sympathetic hyperactivity and reduced cardiac vagal control, which might partly explain the risk of cardiovascular disease in depression [41]. In addition, higher resting HR in depression may also relate to higher anxiety tone and poor night sleep that are often observed during depression, or mild dehydration due to poor oral intake.

Regarding sleep parameters, a total sleep duration of fewer than 6 hours may contribute to the prediction of depressive symptoms, consistent with previous evidence that supported sleep duration remained an important correlate for depressive symptoms [43,44]. For patients who demonstrated a combination of higher resting HR, lower activity, and insufficient sleep, early assessment and intervention of depressive symptoms may be considered.

The successful trial of including individualized features in establishing a machine learning model suggests that it can be potentially applied to other populations, particularly when the focus is on evaluating changes in physiological data or activity levels. For instance, the deviation of HR from baseline may be valuable in predicting events such as panic attacks or aggressive behaviors, where a sudden increase in HR might serve as a critical indicator. The consideration of individualized features is especially relevant in scenarios where baseline data exhibit heterogeneity among individuals, for example, samples comprising both long sleepers and short sleepers. Incorporating individualized features in these contexts can help minimize the risk of biased predictions and enhance the accuracy of the algorithm.

### Limitations

Several limitations need to be addressed. First, the labeling method relies on self-rated questionnaires completed at least once a week which backfill for 7 days given that the questionnaire rated mood symptoms in the past 7 days. While participants were instructed to report mood symptoms as soon as they became aware of them, there is a possibility that some individuals did not complete the questionnaires promptly when their symptoms first emerged. Timely symptom labeling, immediately upon awareness of depressive or manic symptoms, could potentially enhance data reliability. Of note, although the BDI-II is a commonly used self-report measure [45], some studies have shown discrepancies between clinician-rated and self-reported depression severity [46]. Therefore, a clear explanation was provided by the research assistants to the participants to ensure correct understanding of the scales (ie, YMRS and BDI-II) and the use of the mobile app. As for the electronic format of self-rated measures, a recent systematic review focused on the validity of the mobile app–based self-report questionnaires for the assessment and monitoring of BD [47]. Their findings revealed that mobile app–based self-report tools (ie, YMRS, Depression Rating Scale-17, etc) are valid in the assessment of symptoms of mania and depression in patients with BD, indicating good adherence to self-report assessments administered during the study periods.

Second, participants in the study were undergoing treatment with mood stabilizers and/or antipsychotics that may reduce symptom relapse. However, it is essential to acknowledge that medical treatment is inevitable in long-term follow-up in a clinical sample. Future studies may consider recording the days on medications with detailed categorization of psychotropic medications based on their mechanisms to assess their potential impact on sleep and HRs.

Third, low *F*_1_-score in the prediction model for manic symptoms may reflect insufficient events for prediction, which also influences the analysis of feature importance. Meanwhile, the small sample of 24 participants may limit the generalizability of the findings. Although the sample size is not large enough, we followed the participants for 6.39 (SD 4.85) months, thus generating enough nondepressive label (n=1330), depressive label (n=338), nonmanic label (n=1956), and manic label (n=59). A larger sample with a longer follow-up duration may not only ensure enough relapse events for analysis but also increase the generalizability of the findings.

Fourth, potential confounders such as medical treatment, clinical care, interactions, and environmental factors were not controlled in the study. For example, the types and dosing schedule of medications may influence HR and activity level but were not controlled in the analysis, given that all participants received medication treatment with different combinations of antipsychotics and mood stabilizers. Without controlling these factors, the analysis may be incomprehensive and less efficient. Nonetheless, the generated predictive model may be better suited to address the general needs of future applications in real-world scenarios or natural settings whereby participants had no need to provide information other than passive data.

Nevertheless, this study explored the potential of predicting early signs of mood episodes using digital biomarkers. Toward that end, we used the SHAP method to interpret the model and included individualized features that better capture the changes in patients’ lifestyles and successfully improve the prediction models for both depressive and manic symptoms. Our findings highlighted the potential of applying wearable devices to detect early signs of relapse in BD for early intervention. Nowadays, sleep hours, activity level, and HR can be collected by most commercial wearable devices including smartwatches. With an algorithm that can successfully predict upcoming mood episodes and notify the participant, caregiver, or therapist, early assessment and intervention can be initiated in time to prevent a relapse, thus reducing functional impairment and improving the prognosis of BD. Future studies may consider larger, more diverse samples or additional digital biomarkers (eg, environmental data) to enhance the predictive capabilities of the models as well as to explore the efficacy of implementing wearable technology in improving clinical practice and quality of life for various psychiatric populations.

### Conclusions

This study used digital biomarkers obtained from wearable devices to construct machine learning models for the prediction of depressive and manic symptoms. By incorporating individualized features into the models, we achieved satisfactory accuracy in predicting depressive symptoms. Furthermore, the application of an interpretable SHAP model allowed us to discern that higher resting HR, lower activity, and insufficient sleep were indicative of impending depressive symptoms. Early detection of depressive changes enables the timely introduction of adequate psychoeducation and clinical assessment, facilitating the implementation of interventions in time to mitigate upcoming mood episodes, thereby reducing the risk of recurrence.

## Supplementary material

10.2196/66277 Multimedia Appendix 1 Types of psychotropic medications and comparisons of all features between labels.

10.2196/66277 Multimedia Appendix 2 Distribution of Beck Depression Inventory scores.

10.2196/66277 Multimedia Appendix 3 Distribution of Young Mania Rating Scale scores.

## Abbreviations

AUROC: area under the receiver operating characteristic curve
BD: bipolar disorder
BDI: Beck Depression Inventory
*DSM-IV*: *Diagnostic and Statistical Manual of Mental Disorders, Fourth Edition*
HR: heart rate
KNN: k-nearest neighbors
NTU: National Taiwan University
SHAP: Shapley Additive Explanations
SMOTE: Synthetic Minority Over-Sampling Technique
XGBoost: Extreme Gradient Boosting
YMRS: Young Mania Rating Scale
