# Supplementary material for: Using Wearable Device and Machine Learning to Predict Mood Symptoms in Bipolar Disorder: Development and Usability Study
Source: JMIR Med Inform. 2025 Sep 16;13:e66277. doi: 10.2196/66277 (PMC12440259; doi:10.2196/66277)
Supplement: Multimedia Appendix 1 [file medinform-v13-e66277-s001.docx]

**Table S1.** Types of psychotropic medications taken by the participants.

| Medications | N | % |
| --- | --- | --- |
| Valproic acid | 11 | 45.83 |
| Lithium | 11 | 45.83 |
| Quetiapine | 12 | 50.00 |
| Long-acting injection | 3 | 12.50 |
| Carbamazepine | 1 | 4.17 |
| Haloperidol | 4 | 16.67 |
| Trifluoperazine | 1 | 4.17 |
| Risperidone | 3 | 12.50 |
| Lurasidone | 3 | 12.50 |
| Amisulpiride | 1 | 4.17 |
| Paliperidone | 1 | 4.17 |
| Wellbutrin | 3 | 12.50 |
| Lamotrigine | 1 | 4.17 |
| Sertraline | 1 | 4.17 |
| Mirtazapine | 1 | 4.17 |
| Vortioxetine | 1 | 4.17 |

Table S2. Comparisons of all features between depressive and non-depressive labels.

|  | **Non-depressive** **label** (data n = 1,330)  Mean (SD) | **Depressive** **label** (data n = 338)  Mean (SD) | t values |
| --- | --- | --- | --- |
| Min heart rate (bpm) | 53.28 (8.47) | 57.11 (7.09) | 7.67* |
| Max heart rate (bpm) | 125.10 (17.67) | 123.70 (15.58) | -1.33 |
| Average heart rate (bpm) | 75.55 (10.26) | 76.54 (8.39) | 1.64 |
| Resting heart rate (bpm) | 59.50 (9.66) | 64.01 (7.03) | 8.05* |
| Steps (thousand) | 7.77 (5.33) | 5.98 (4.43) | -5.69* |
| Distance (meter) | 5,364 (3,816) | 4,018 (3,028) | -6.02* |
| Floors climbed | 5.78 (10.10) | 4.66 (4.82) | -1.98 |
| Total sleep duration (hour) | 8.45 (2.07) | 8.44 (2.25) | -0.03 |
| Deep sleep duration (hour) | 1.46 (1.37) | 2.09 (1.56) | 7.32* |
| Light sleep duration (hour) | 5.30 (1.77) | 5.12 (1.82) | -1.70 |
| REM sleep duration (hour) | 1.64 (1.06) | 1.22 (0.84) | -6.75* |
| Awake duration (hour) | 0.16 (0.23) | 0.23 (0.31) | 4.60* |
| **Individualized features** |  |  |  |
| Min heart rate (bpm) | -0.04 (4.19) | 0.17 (5.00) | 0.78 |
| Max heart rate (bpm) | -0.01 (13.82) | 0.05 (12.92) | 0.08 |
| Average heart rate (bpm) | 0.02 (4.99) | -0.07 (5.32) | -0.27 |
| Resting heart rate (bpm) | -0.08 (4.18) | 0.32 (4.31) | 1.59 |
| Steps (thousand) | -0.02 (4.35) | 0.07 (3.67) | 0.36 |
| Distance (meter) | -7.07 (3,073) | 27.82 (2,555) | 0.19 |
| Total sleep duration (hour) | 0 (1.73) | 0.02 (1.89) | 0.23 |

Note. REM, rapid eye movement, *p < 0.05

Table S3. Comparisons of features between manic and non-manic labels.

|  | **Non-manic label** (data n = 1,956)  Mean (SD) | **Manic** **label** (data n = 59)  Mean (SD) | t values |
| --- | --- | --- | --- |
| Min heart rate (bpm) | 54.60 (8.78) | 54.71 (9.07) | 0.10 |
| Max heart rate (bpm) | 124.31 (16.56) | 123.5 (17.06) | -0.33 |
| Average heart rate (bpm) | 76.30 (10.51) | 76.86 (11.23) | 0.40 |
| Resting heart rate (bpm) | 61.03 (9.96) | 61.80 (9.93) | 0.58 |
| Steps (thousand) | 7.79 (5.27) | 6.76 (3.58) | -1.50 |
| Distance (meter) | 5,334 (3,737) | 4,523 (2,416) | -1.66 |
| Floors climbed | 6.14 (9.46) | 5.19 (6.28) | -0.77 |
| Total sleep duration (hour) | 8.43 (2.05) | 7.88 (2.43) | -2.02* |
| Deep sleep duration (hour) | 1.73 (1.55) | 2.31 (0.96) | 2.82* |
| Light sleep duration (hour) | 5.18 (1.77) | 4.35 (1.67) | -3.57* |
| REM sleep duration (hour) | 1.48 (1.03) | 1.19 (0.81) | -2.15* |
| Awake duration (hour) | 0.17 (0.24) | 0.13 (0.15) | -1.23 |
| **Individualized features** |  |  |  |
| Min heart rate (bpm) | 0 (4.50) | 0.06 (5.05) | 0.10 |
| Max heart rate (bpm) | -0.07 (12.66) | 2.27 (13.50) | 1.39 |
| Average heart rate (bpm) | -0.09 (4.81) | 2.96 (5.07) | 4.78* |
| Resting heart rate (bpm) | -0.02 (4.37) | 0.72 (5.73) | 1.28 |
| Steps (thousand) | -0.04 (4.21) | 1.27 (3.04) | 2.37* |
| Distance (meter) | -26.78 (2,931) | 887.89 (2,088) | 2.38* |
| Total sleep duration (hour) | 0.02 (1.75) | -0.79 (2.18) | -3.50* |

Note. REM, rapid eye movement, *p < 0.05
