# Supplementary figures and images for: Using Wearable Device and Machine Learning to Predict Mood Symptoms in Bipolar Disorder: Development and Usability Study
Source: JMIR Med Inform. 2025 Sep 16;13:e66277. doi: 10.2196/66277 (PMC12440259; doi:10.2196/66277)

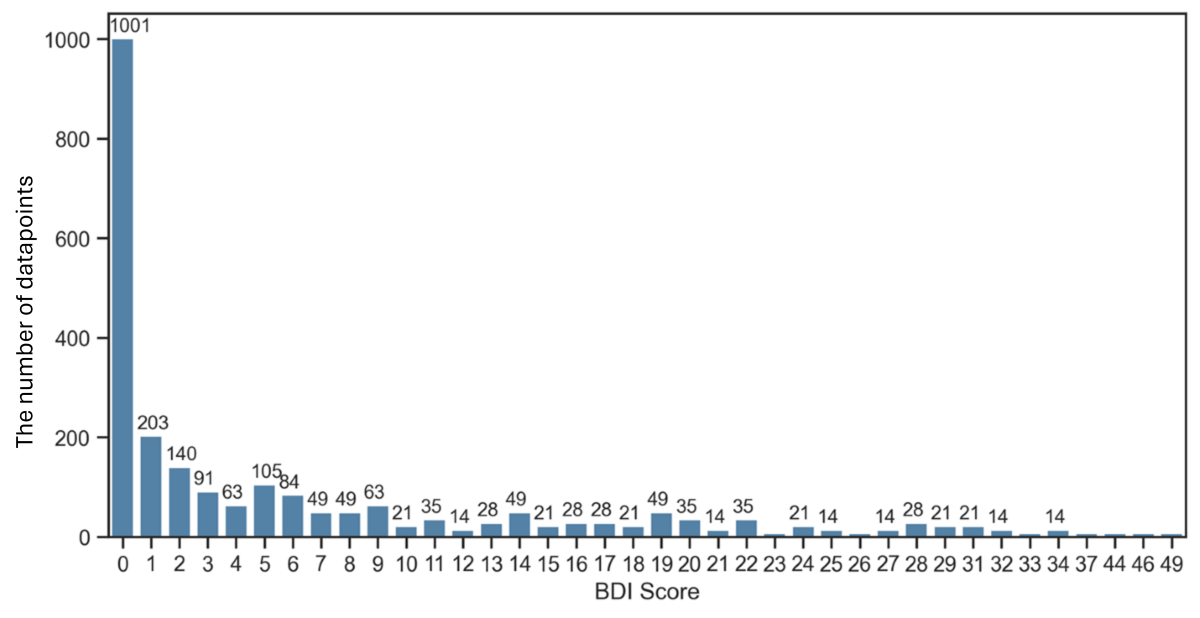

Supplement: Multimedia Appendix 2 [file medinform-v13-e66277-s002.png]

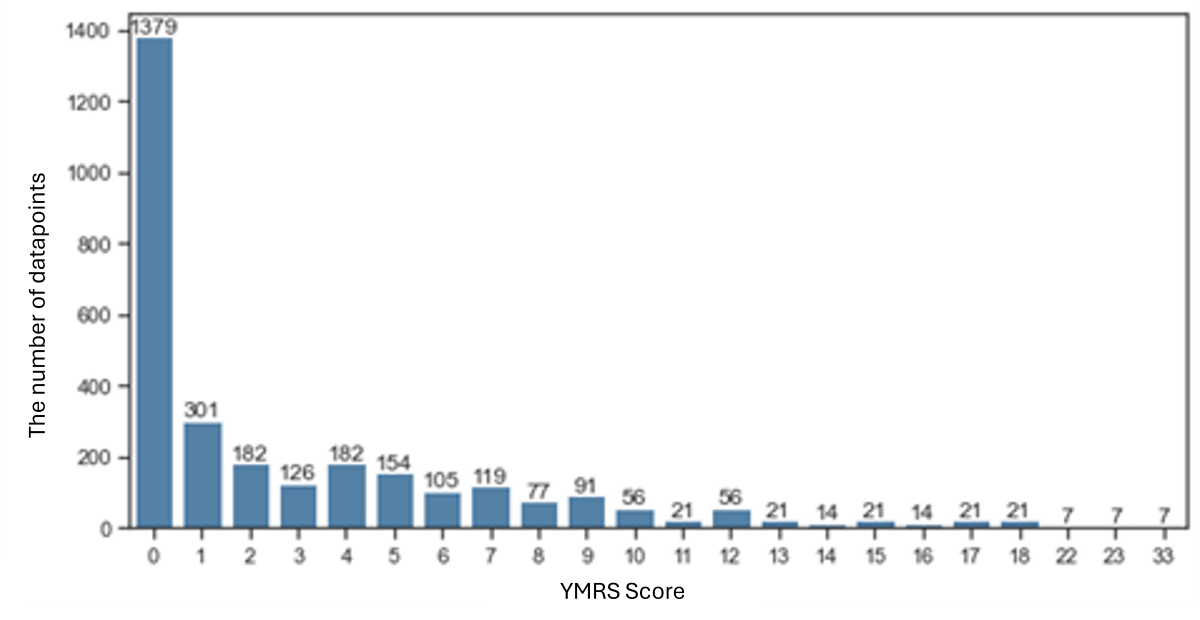

Supplement: Multimedia Appendix 3 [file medinform-v13-e66277-s003.png]
